# Proteomics-based functional studies reveal that galectin-3 plays a protective role in the pathogenesis of intestinal Behçet’s disease

**DOI:** 10.1038/s41598-019-48291-1

**Published:** 2019-08-12

**Authors:** Hyun Jung Lee, Jae Hyeon Kim, Sujeong Hong, Inhwa Hwang, Soo Jung Park, Tae Il Kim, Won Ho Kim, Je-Wook Yu, Seung Won Kim, Jae Hee Cheon

**Affiliations:** 1 Department of Internal Medicine and Institute of Gastroenterology, Yonsei University College of Medicine, Seoul, Korea; 2 Department of Internal Medicine and Liver Research Institute, Seoul National University College of Medicine, Seoul, Korea; 3 Brain Korea 21 PLUS Project for Medical Science, Yonsei University College of Medicine, Seoul, Korea; 4 Department of Microbiology, Institute for Immunology and Immunological Diseases, Brain Korea 21 PLUS Project for Medical Science, Yonsei University College of Medicine, Seoul, Korea; 5 Severance Biomedical Science Institute, Yonsei University College of Medicine, Seoul, Korea

**Keywords:** Immunology, Inflammation

## Abstract

The pathogenesis of intestinal Behçet’s disease (BD) remains poorly understood. Therefore, we aimed to discover and validate biomarkers using proteomics analysis and subsequent functional studies. After two-dimensional electrophoresis, candidate proteins were identified using matrix-assisted laser desorption/ionization tandem time-of-flight mass spectrometry (MALDI-TOF/TOF MS). We validated these results by evaluating the protein levels and their functions *in vitro* using HT-29 colorectal cancer cells, colon tissues from patients and mice, and murine bone marrow derived macrophages (BMDMs). Of the 30 proteins differentially expressed in intestinal BD tissues, we identified seven using MALDI-TOF/TOF MS. Focusing on galectin-3, we found that *TGF-B* and *IL-10* expression was significantly lower in shLGALS3-transfected cells. Expression of *GRP78* and *XBP1s* and apoptosis rates were all higher in shLGALS3-transfected cells upon the induction of endoplasmic reticulum stress. In response to lipopolysaccharide stimulation, microtubule-associated protein 1 light chain 3B accumulated and lysosomes decreased in these cells. Finally, *Salmonella typhimurium* infection induced caspase-1 activation and increased IL-1β production, which facilitated activation of the NLRC4 inflammasome, in *Lgals3*^−/−^ murine BMDMs compared to wild type BMDMs. Our data suggest that galectin-3 may play a protective role in the pathogenesis of intestinal BD via modulation of ER stress, autophagy, and inflammasome activation.

## Introduction

Intestinal Behçet’s disease (BD) is a chronic, relapsing inflammatory disorder of the gastrointestinal tract; the diagnosis of this disease is typically based on colonoscopic features and clinical manifestations^1^. The incidence of intestinal BD is higher in East Asian countries, including Korea and Japan, compared to the Middle Eastern countries^2^. Intestinal BD has an unpredictable disease course with exacerbation and remission, and often requires surgical treatment due to massive bleeding, fistula, or bowel perforation^3,4^. Because the surgery rates for intestinal BD are comparable to those for Crohn’s disease (CD) due to high rates of complications^5^, better methods for diagnosis and management of intestinal BD are needed. A better understanding of the pathogenesis of intestinal BD would be invaluable.

Both genetic and environmental factors contribute to inflammatory bowel disease (IBD) etiology. A growing body of evidence demonstrates that epithelial barrier defects and dysregulated mucosal immune responses to intestinal microbes in genetically susceptible individuals might result in sustained intestinal inflammation^6^. Dysregulated enterocyte shedding and apoptosis is known to cause intestinal barrier instability^7^ and inappropriate innate immune responses to commensal flora and defective autophagy function are observed in patients with IBD^8,9^. To date, genome-wide association studies (GWAS) have revealed more than 200 genetic biomarkers for the development of IBD, including *CARD15/NOD2*, *ATG16L1*, and *IRGM*^8–11^. In general, intestinal BD has similar clinical and therapeutic characteristics to IBD. However, although we recently identified several genetic loci (*IL17A*, *IL23R*, *STAT4, NAALADL2*, and *YIPF7*) associated with susceptibility to intestinal BD^12,13^, relatively little is known about the disease pathogenesis of intestinal BD, highlighting the need for further study to aid in the diagnosis and treatment of this disease.

In recent decades, because of the limitations of currently available biomarkers, proteomics is gaining popularity for biomarker discovery in IBD. These approaches have proven particularly useful for the identification of IBD biomarkers, aiding in diagnosis, measurement of disease activity, and the prediction of treatment response^14–16^. In contrast, to the best of our knowledge, a proteomics approach to identify biomarkers in intestinal BD has not yet been described. Therefore, we aimed to find biomarkers associated with intestinal BD pathogenesis using two-dimensional gel electrophoresis (2-DE) and mass spectrometry (MS)-based proteomics. Furthermore, we sought to elucidate the pathogenic mechanisms of a single candidate protein using various *in vitro* functional studies. Our work, for the first time, describes the proteomics approaches as useful techniques for identifying biomarkers associated with intestinal BD and reports a previously undescribed association between the disease and the galectin-3 (Gal-3) protein, suggesting a potential significance of this protein in the pathogenesis of intestinal BD.

## Methods

### Human subject demographics and sample collection

A total of 17 patients diagnosed with intestinal BD and 17 non-affected control individuals were enrolled in this study at Severance Hospital, Yonsei University, in Seoul, Korea. Intestinal BD diagnosis was made according to previously established criteria based on colonoscopic features and clinical manifestations using a modified Delphi process^1^. Tissue samples were obtained from both inflamed and non-inflamed intestinal tissues of intestinal BD patients undergoing surgery due to bowel complications and/or intractability to medical treatments. Control samples were obtained from normal intestinal tissues from subjects undergoing colorectal surgery for colorectal cancer, malignant bowel obstruction due to stomach cancer, or appendiceal mucocele. Samples were immediately frozen in liquid nitrogen and subsequently stored at −80 °C until preparation for proteomic analyses.

Informed consent was obtained from all individuals enrolled in this study. This study was approved by the Institutional Review Boards of Yonsei University College of Medicine (IRB approval number: 4-2012-0680) and was conducted in accordance with the Declaration of Helsinki.

### Two-dimensional electrophoresis (2-DE)

Two-dimensional electrophoresis (2-DE) was carried out essentially as described^17,18^. Aliquots in sample buffer (7 M urea, 2 M thiourea, 4.5% CHAPS, 100 mM DTT, 40 mM Tris, pH 8.8) were applied to immobilized pH 3–10 nonlinear gradient strips (Amersham Biosciences, Uppsala, Sweden). Isoelectric focusing (IEF) was performed at 80,000 Vh. The second dimension was analyzed on 9–16% linear gradient polyacrylamide gels (18 cm × 20 cm × 1.5 mm) at a constant 40 mA *per* gel for approximately 5 h. After protein fixation in 40% methanol and 5% phosphoric acid for 1 h, the gels were stained with Coomassie G-250 solution for 12 h. The gels were destained with H_2_O, scanned in a Bio-Rad GS710 densitometer (Life Science, Richmond, CA, USA) and converted into electronic files, which were then analyzed with Image Master Platinum 5.0 (Amersham Biosciences).

### Protein identification by MALDI‒TOF/TOF MS

Protein spots were excised from the gels with a sterile scalpel and placed into Eppendorf tubes. Proteins were digested using trypsin (Promega, Madison, WI, USA) as previously described^17,19^. For matrix-assisted laser desorption/ionization tandem time-of-flight mass spectrometry (MALDI‒TOF/TOF MS) analysis, the tryptic peptides were concentrated by a POROS R2, Oligo R3 column (Applied Biosystems, Foster City, CA, USA). After washing the column with 70% acetonitrile, 100% acetonitrile and then 50 mM ammonium bicarbonate, samples were applied to the R2, R3 column and eluted with cyano-4-hydroxycinamic acid (CHCA) (Sigma-Aldrich, St. Louis, MO, USA), and then dissolved in 70% acetonitrile and 0.1% TFA before MALDI-TOF/TOF MS analysis. Mass spectra were acquired on a 4800 Proteomics Analyzer (Applied Biosystems) operated in MS and MS/MS modes. Peptide fragmentation in MS/MS mode was by collision-induced dissociation (CID) using atmosphere as the collision gas. The instrument was operated in reflectron mode and calibrated using the 4700 calibration mixture (Applied Biosystems) and each sample spectrum was additionally calibrated using trypsin autolysis peaks. For MS analysis, a 800–4000 *m/z* mass range was used with 1000 shots per spectrum. A maximum of 15 precursors with a minimum S/N of 50 were selected for MS/MS analysis. Collision energy of 1 kV was used for CID, and 2000 acquisitions were accumulated for each MS/MS spectrum. Peptide mass fingerprinting was carried out using the Mascot search engine included in the GPS Explorer software and mass spectra used for manual *de novo* sequencing were annotated with the Data Explorer software (Applied Biosystems).

### Mascot database search

The mascot algorithm (Matrixscience, Boston, MA, USA) was used to identify peptide sequences present in a protein sequence database as previously described^17^. Database search criteria were, taxonomy; *homo sapiens* (NCBInr database downloaded on Mar 24 2013), fixed modification; carboxyamidomethylated (+57) at cysteine residues; variable modification; oxidized (+16) at methionine residues, maximum allowed missed cleavage, 1. Mass tolerances of 100 ppm, 0.1 Da were used for precursor and fragment ions, respectively. Only peptides resulting from trypsin digests were used for protein identification.

### Immunohistochemical staining of colon tissues to verify 2-DE and MALDI-TOF results

We performed immunohistochemistry (IHC) staining on tissue sections to quantify Gal-3 levels using a Vecastain ABC kit (Vector Labs, Burlingame, CA, USA). Tissue sections were incubated first with the primary anti-Gal-3 antibody (1:200, Santa Cruz Biotechnology, Inc., Santa Cruz, CA, USA) overnight at 4 °C, followed by incubation with a biotinylated secondary linking antibody for 1 h, and finally for 1 h with a streptavidin-peroxidase complex. The final color product was developed using aminoethylcarbazole (Dako, Carpinteria, CA, USA). Sections were counterstained with hematoxylin and mounted, and the tissues were photographed using an Olympus photomicroscope (Olympus Corp., Tokyo, Japan). For quantitative analysis, we randomly selected 4 fields for each sample at 200× magnification and scored the ratios of positively Gal-3 stained cells to all cells. The percentage of positive cells and the intensity of staining were scored from 0 to 3 (0 =< 10%, 1 = 10–50%, 2 = 50–75%, 3 = 75–100%) as previously described^20^.

### Western blotting

Proteins were extracted from colon tissues or HT-29 cells and were lysed and homogenized in a buffer containing 50 mM Tris-Cl at pH 8.0, 150 mM NaCl, 0.1% SDS, 1 mM EDTA, 1% Triton‒X, 0.05% sodium deoxycholate, and protease inhibitors. Protein samples were fractionated on 12% SDS-PAGE and transferred onto polyvinylidene difluoride (PVDF) membranes for 70 min at 100 volts (Bio-Rad). Blots were incubated with primary anti-Gal-3 antibody (1:2000, sc-20157, Santa Cruz Biotechnology, Inc., Santa Cruz, CA, USA), anti-caspase-1 antibody (1:2000, Santa Cruz), anti-GRP78 antibody (1:2000, ABCAM, Cambridge, MA, USA), anti-XBP-1 antibody (1:2000, NOVUS BIO, Littleton, CO, USA), and anti-IL-1β antibody (1:2000, R&D system, MN, USA) followed by horseradish peroxidase-conjugated secondary antibody and enhanced chemiluminescence (ECL) reagents using the ECL kit (Thermo Scientific, MA, USA). Anti-β-actin (Santa Cruz Biotechnology) was used as the loading control.

### Quantitative real-time reverse-transcription polymerase chain reaction (qRT-PCR)

Total RNA extraction and reverse-transcription was performed as previously described^17,21^. PCR primer information is described in Supplementary Table 1. Samples were amplified in a StepOne Plus real-time PCR system (Applied Biosystems) for 45–55 cycles using the following cycling conditions: 95 °C for 15 sec, 60–63 °C for 30 sec, and 72 °C for 40 sec. Quantitative analysis was performed using the relative standard curve, and the results were reported as a relative expression or fold change compared to the calibrator after normalization of the transcript level against control, *β-ACTIN*.

### Transfection and treatment of HT-29 cells

We maintained the HT-29 colon cancer cell line (KCLB 30038, Korean Cell Line Bank, Seoul, Korea) at 37 °C in Roswell Park Memorial Institute medium (RPMI medium) supplemented with 10% heat-inactivated fetal bovine serum (FBS) and 1% antibiotics in a humidified atmosphere of 5% CO_2_. We transfected HT-29 cells with human Gal-3 small hairpin RNA (shRNA, Santa Cruz Biotechnology) plasmids to create Gal-3 knock-down cell lines using Lipofectamine 2000 (Invitrogen, Carlsbad, CA, USA). Control cells with transfected with scrambled shRNA. Successfully transfected cells were isolated by selection with 20 μg/ml puromycin (Santa Cruz Biotechnology) for 4 weeks. After purification of Puromycin-resistant cell lines, Gal-3 expression was evaluated using Western blot analysis. Subsequently, stable Gal-3-silenced cell lines (shLGALS3) and control cell lines (SCR, scramble) were selected for further analysis. Cells were incubated with TNF-α (40 ng/mL, R&D systems) and lipopolysaccharide (LPS, 1–10 μg/mL, Sigma-Aldrich) with or without recombinant human Gal-3 (10 μM, Prospec, NJ, USA) for 4 h (qRT-PCR) or 24 h (western blot).

### Immunofluorescence staining to quantify Gal-3 and LC3B protein levels and trace lysosomes in transfected HT-29 cells

To quantify Gal-3 protein levels in shLGALS3 transfected and control HT-29 cells we performed immunofluorescence staining as follows: First, cells were fixed with a 4% paraformaldehyde solution (pH 7.4). After a PBS wash, cells were blocked in 5% BSA with 0.1% Triton X-100, washed with PBS again, and incubated with primary antibodies (anti-microtubule-associated protein 1 light chain‒3B (LC3B), 1:1,000; anti-Gal-3, 1:500). Primary antibodies were fluorescently labeled with Alexa Fluor 488- and 633-conjugated secondary antibodies (1:500). Cell nuclei were stained with DAPI solution. For tracing lysosomal fusion with autophagosomes, cells were incubated with LysoTracker^®^ Red DND-99 (100 nM, Molecular Probes, Eugene, OR, USA). Images were obtained via fluorescence microscopy (Olympus BX41; Olympus Optical, Tokyo, Japan) at a magnification of 400× or confocal microscopy (Carl Zeiss LSM 700, Prenzlauer, Berlin, Germany) at a magnification of 800×.

### Quantification of apoptosis in shLGALS3-transfected HT-29 cells via flow cytometry

Apoptosis in transfected HT-29 cells were quantified via flow cytometry using an Annexin V-FITC/PI kit (BD Biosciences, San Diego, CA, USA). A total of 30,000 events were collected from each sample using a FACSverse flow cytometer (BD Biosciences), and the resulting spectra were analyzed using Flow Jo software (Tree Star, San Carlos, CA, USA).

### Isolation of bone marrow-derived murine macrophages

Murine primary bone marrow-derived macrophages (BMDMs) were prepared from C57BL/6 mice as described previously^22^. Bone marrow cells were isolated from femurs of C57BL/6 mice and differentiated for one week in L929-conditioned DMEM/F-12 medium supplemented with 10% FBS. All mice were maintained under specific pathogen-free conditions, and all experiments using BMDMs were performed in accordance with the approved guidelines of the Institutional Animal Care and Use Committee of Yonsei University Severance Hospital, Seoul, Korea (IACUC, Approval No: 2013–0166). All BMDMs were maintained in L929-conditioned DMEM supplemented with 10% FBS and 100 U/mL penicillin/streptomycin.

### Statistical analysis

Data are expressed as mean ± standard deviation (SD) or ± standard error of mean (SEM). Parametric and nonparametric analyses were performed using Student’s *t*-test and Mann-Whitney *U* test, respectively. *P* < 0.05 was considered statistically significant. All statistical analyses were performed using SPSS V20.0 for Windows (SPSS Inc., Chicago, IL, USA) or Prism software 5 (GraphPad Software, Inc., San Diego, CA, USA).

## Results

### Gal-3 is down-regulated in inflamed intestinal tissue from intestinal BD patients

The clinical characteristics of 17 intestinal BD cases and 17 non-affected controls enrolled in this study are shown in Supplementary Table 2.

**Figure 1:**
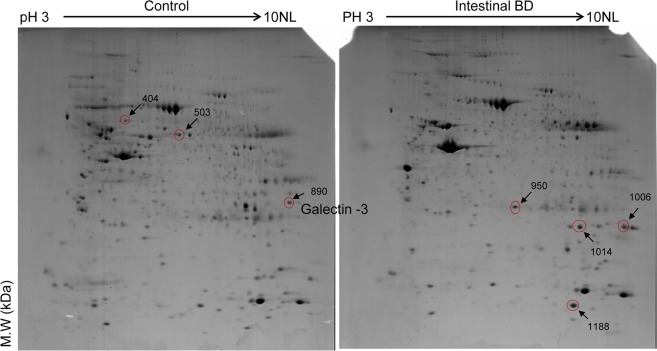
Proteomic profiles of intestinal tissue from patients with intestinal Behçet’s disease and non-affected controls. Seven significantly differentially expressed protein fractions (red circles) identified via two-dimensional electrophoresis (2-DE) were excised and further analyzed by matrix-assisted laser desorption/ionization tandem time-of-flight mass spectrometry (MALDI-TOF/TOF MS) (arrows on 2-DE gels). Spot number was assigned by image analysis program, followed by 2-DE.

We first performed a tissue proteomics analysis using 2-DE to identify differentially expressed proteins between the case and control groups. Approximately 550 protein spots were observed (Fig. 1), with a total of 30 protein spots found to have qualitative or quantitative differences in intestinal BD tissues compared to controls. Specifically, 12 were up-regulated, 15 were down-regulated, and 3 were observed in the intestinal BD tissues alone. We focused on highly differently expressed proteins only; therefore, spots with a 5-fold or higher change between case and control tissues and uniquely manifested in either of the two gels were defined as differential. Seven (four upregulated and three downregulated) of these highly differentially expressed proteins were incised from the gels and analyzed by MALDI-TOF/TOF MS for protein identification. The upregulated proteins were identified as heat shock protein 27 (2.2-fold), transgelin (4.7-fold), superoxide dismutase (3.3-fold), and calprotectin (9.3-fold). The down-regulated proteins were identified as heat shock protein 60 (−7.0-fold), selenium binding protein (−9.0-fold), and Gal-3 (−5.8-fold) (Table 1). These proteins are known to be involved in stress response, oxidative stress, and/or inflammation^23–25^. Interestingly, Gal-3 has been reported as an immune response regulator in chronic inflammatory disorders including IBD^26–28^. Therefore, we focused on Gal-3 for further investigations. The typical peptide mass fingerprinting (PMF) and MS/MS spectrum of Gal-3 are shown in Supplementary Fig. 1.

**Table 1:** Protein identification by MALDI-TOF/TOF.

Spot no	Protein description	Accession no	Mr(kDa)/p*I*	Matched peptide sequence	Fold change
404	60 kDa heat shock protein, mitochondrial	gi|129379	61187/5.70	IGIEIIKR(41) TVIIEQSWGSPK(79) GYISPYFINTSK(57) VGEVIVTKDDAMLLK(17) ISSIQSIVPALEIANAHR(28) IQEIIEQLDVTTSEYEKEK(37) KPLVIIAEDVDGEALSTLVLNR(95) DMAIATGGAVFGEEGLTLNLEDVQPHDLGK(4)	−7.0
503	Selenium binding protein1	gi|14290607	52928/5.93	DGLIPLEIR(41) LVLPSLISSR(18) HEIVQTLSLK(87) IYVVDVGSEPR(51) QYDISDPQRPR(30) GGFVLLDGETFEVK(35) VAGGPQMIQLSLDGKR(32) HNVMISTEWAAPNVLR(33) GTWERPGGAAPLGYDFWYQPR(22) DGFNPADVEAGLYGSHLYVWDWQR(38)	−9.0
890	Galectin-3	gi|2385452	26193/8.57	IALDFQR(35) FNENNRR(35) IQVLVEPDHFK(72) QSVFPFESGKPFK(90) MLITILGTVKPNANR(26) LGISGDIDLTSASYTMI(10)	−5.8
950	Heat shock protein 27	gi|11036357	22826/5.98	VPFSLLR(52) AQLGGPEAAK(37) DWYPHSR(44) RVPFSLLR(26) QLSSGVSEIR(90) QDEHGYISR(61) HEERQDEHGYISR(46) VSLDVNHFAPDELTVK(108)	2.2
1006	Transgelin	gi|3123283	22653/8.87	GDPNWFMK(31) QMEQVAQFLK(25) VPENPPSMVFK(64) VPENPPSMVFK(27) GASQAGMTGYGRPR(20) LVNSLYPDGSKPVK(79) TDMFQTVDLFEGK(67) TDMFQTVDLFEGKDMAAVQR(134) TDMFQTVDLFEGKDMAAVQR(43) AAEDYGVIKTDMFQTVDLFEGK(30)	4.7
1014	Superoxidedismutase	gi|134665	24878/8.35	NVRPDYLK(41) GELLEAIKR(58) DFGSFDKFK(67) GDVTAQIALQPALK.(103) LTAASVGVQGSGWGWLGFNK.(155) HHAAYVNNLNVTEEKYQEALAK(44)	3.3
1188	Protein S100-A8	gi|115442	10885/6.51	MGVAAHKK(14) GNFHAVYR(60) GNFHAVYRDDLK(50) ELDINTDGAVNFQEFLILVIK(60)	9.3

Mr, molecular mass (kDa).

**Figure 2:**
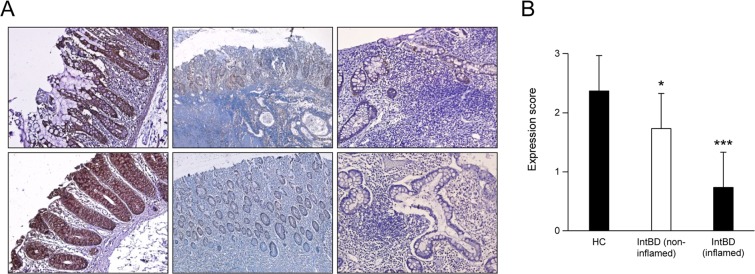
Galectin‒3 is down-regulated in inflamed intestinal tissues from intestinal Behçet’s disease patients. (**A**,**B)** Immunohistochemistry of galectin-3. Intestinal tissues from non-affected controls (n = 15) and patients with intestinal Behçet’s disease (n = 15) were stained with anti-galectin-3 antibodies and counterstained with hematoxylin. Representative images of inflamed and non-inflamed intestinal tissues are shown in **(A)** at 200 × magnification. Galectin-3 expression in normal, inflamed, and non-inflamed intestinal tissues is shown in **(B)**. Results are shown as individual values (symbols) with the standard deviation indicated (lines) for each group of patients. ^*^p < 0.05 vs. HC, ^***^p < 0.005 vs. HC. HC, healthy control; intBD, intestinal BD.

We next used IHC staining of the colonic tissues to confirm whether Gal-3 levels are lower in intestinal BD. IHC confirmed that Gal-3 levels were significantly reduced in both the inflamed and non-inflamed crypt epithelia from intestinal BD patients compared to non-inflamed control tissues (Fig. 2A,B). Therefore, we hypothesized that Gal-3 may play a protective role against development of intestinal BD, and that when Gal-3 expression is decreased in the inflamed intestinal tissues, an individual is at risk for developing intestinal BD. Taken together, these data support our proteomic observation that Gal-3 might play a protective role and be involved in the pathogenesis of intestinal BD. When it is down-regulated in the inflamed intestinal mucosa, intestinal BD can develop.

### Gal-3 knockdown negatively affects anti-inflammatory cytokine expressions in intestinal epithelial cells

**Figure 3:**
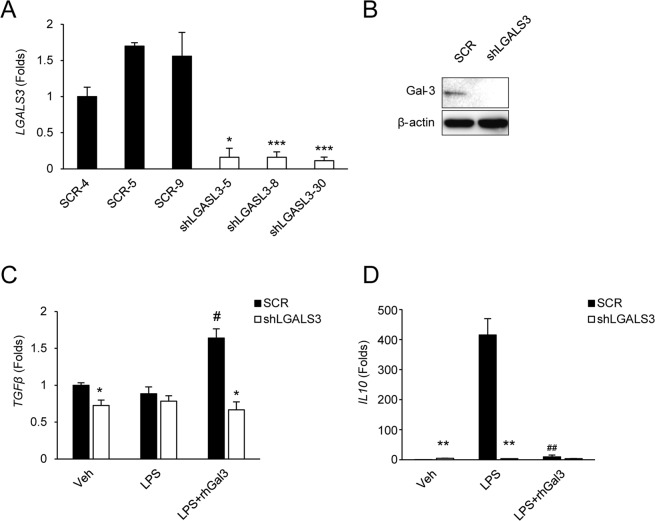
Loss of galectin-3 affects anti-inflammatory cytokine expression in HT-29 cells. (**A)** Transcript levels of galectin-3 gene (*LGALS3*). (**B**) Galectin-3 protein levels. Transcript levels of **(C)** *TGF-B* and **(D)** *IL-10*. Data are presented as the mean ± SD (n = 3). ^*^p < 0.05 vs. SCR, ^**^p < 0.01 vs. SCR, ^***^p < 0.05 vs. SCR, ^#^*p* < 0.05 vs. LPS, ^##^*p* < 0.01 vs. LPS. Veh, treated with phosphate-buffered saline; LPS, treated with lipopolysaccharide; rhGal3, treated with recombinant human galectin-3; SCR, scramble control HT-29 cell; shLGALS3, HT-29 cell stably expressing LGALS3 shRNA.

Because Gal-3 is a reported immune response regulator, we next determined whether Gal-3 could affect anti-inflammatory cytokine expression in human HT-29 colorectal cancer cells. We knocked-down the *LGALS3* gene in HT-29 cells via shRNA transfection (shLGALS3) and established a stable cell line through antibiotics selection (Supplementary Fig. 2). We confirmed decreased *LGALS3* levels using quantitative RT-PCR and Western blotting (Fig. 3A,B and Supplementary Fig. 3A,B, respectively). When the transfected cells were treated with LPS to induce an inflammatory response, the expression of anti-inflammatory cytokines *TGF-β* and *IL-10* was significantly lower in cells transfected with shLGALS3 compared to scramble-transfected cells (Fig. 3C,D), confirming the role of Gal-3 as an immune response regulator in intestinal epithelial cells.

### Loss of Gal-3 leads to endoplasmic reticulum stress-induced cell death and induces autophagy defects in intestinal epithelial cells

**Figure 4:**
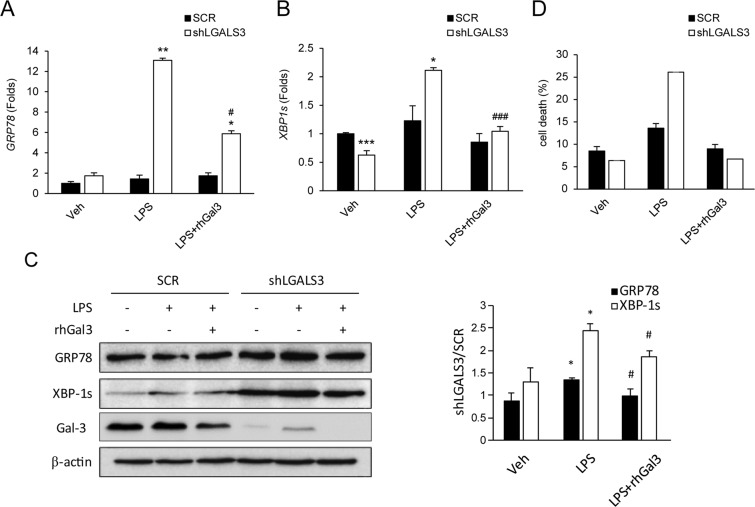
Loss of galectin-3 activates endoplasmic reticulum (ER) stress and promotes cell death in HT-29 cells. (**A**,**B)** Expression of ER and cellular stress indicator proteins *GRP78* and *XBP1s* in cells treated with LPS (**A,B**). Transcript levels were quantified by real-time quantitative reverse transcription polymerase chain reaction and normalized to *β-actin* expression levels. **(C)** Protein levels were quantified by western blotting and normalized to *β-actin*. ^*^p < 0.05 vs. SCR, ^**^p < 0.01 vs. SCR, ^***^p < 0.05 vs. SCR, ^#^*p* < 0.05 vs. LPS, ^###^*p* < 0.005 vs. LPS. Veh, treated with phosphate-buffered saline; LPS, treated with lipopolysaccharide; rhGal3, treated with recombinant human galectin-3; SCR, scramble control HT-29 cell; shLGALS3, HT-29 cell expressing stably LGALS3 shRNA. **(D)** Effects of galectin-3 on cell death by ER stress inducer, LPS, were shown through Annexin V/PI staining. Data are presented as mean ± SD (n = 3). Veh, treated with phosphate-buffered saline; LPS treated with lipopolysaccharide; rhGal3, treated with recombinant human galectin-3 (10 μM); SCR, scramble control HT-29 cell; shLGALS3, HT-29 cell stably expressing LGALS3 shRNA.

Recent studies have reported that unresolved endoplasmic reticulum (ER) stress and autophagy defects can trigger epithelial cell apoptosis and inflammation^29,30^, and that these defects are linked to impaired barrier function and IBD pathogenesis^7^. Therefore, we determined whether similar defects were present in intestinal epithelial cells lacking Gal-3, which would support a role for Gal-3 in preventing intestinal BD-associated autophagy defects and ER stress-associated cell death. We induced ER stress in shLGALS3 cells using LPS, thapsigargin, or serum starvation. Expression of the ER and cellular stress-associated glucose-regulated protein (*GRP78*) and spliced *XBP1* (*XBP1s*), an active spliced form of X-box–binding protein 1, were markedly increased by LPS-stimulation in shLGALS3 cells (Fig. 4A–C and Supplementary Fig. 4A–D). Additionally, ER stress by all three conditions was associated with a significantly higher incidence of cell death in shLGALS3 cells, and this increased cell death was reversed upon treatment with Gal-3 (Fig. 4D and Supplementary Fig. 5A,B).

**Figure 5:**
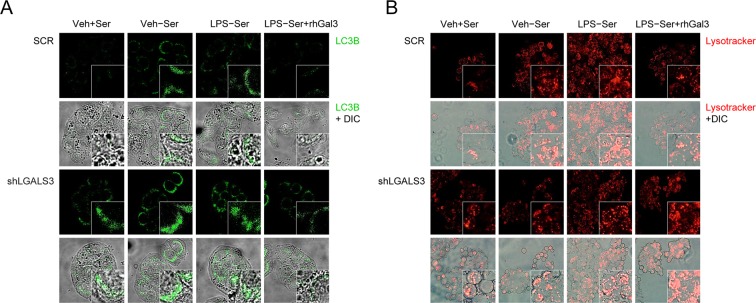
Loss of galectin-3 induces autophagy defects. Transfected HT-29 cells were treated with LPS to induce ER stress and subsequent autophagy. **(A)** Representative confocal images of autophagosomic endogenous LC3B localization. **(B)** Representative confocal images of lysosomes at 800 × magnification. Red color indicates LysoTracker, an indicator of phagosomal acidification. Experiments were performed in triplicate. Veh, treated with phosphate-buffered saline; +Ser, 10% serum supplemented; -Ser, serum free; LPS, treated with lipopolysaccharide; rhGal3, treated with recombinant human galectin-3 (10 μM); SCR, scramble control HT-29 cells; shLGALS3, HT-29 cells stably expressing LGALS3 shRNA; DIC, differential interference contrast (DIC).

Because Gal-3 has been proposed as a marker of lysed vacuole membranes and a target of autophagy^31^, we next investigated the role of Gal-3 in the process of autophagic removal of invasive pathogens. Treatment of HT-29 cells with a TLR4 agonist, LPS, led to a significant increase in autophagosomes, as demonstrated by an increase in the detection of LC3B, a central protein in the autophagy pathway, within cells by fluorescence microscopy. Autophagic cell numbers were increased in abundance among the shLGALS3 cell population in response to serum starvation and LPS, as reflected by increased accumulation of LC3B-positive vesicles (punctae, Fig. 5A). Additionally, there were more apoptotic bodies in shLGALS3 cells compared to scramble shRNA-transfected control cells (Supplementary Fig. 6A). In contrast, phagosomal acidification was lower in shLGALS3 cells upon serum starvation and LPS compared to control cells (Fig. 5B and Supplementary Fig. 6B). Notably, LC3B was not recruited to Gal-3 puncta, but co-localization of Gal-3 with lysosomes increased in response to serum starvation and LPS, which might result in decreased autolysosome formation (Supplementary Fig. 7A–C). Altogether, our data suggest that Gal-3 plays a role in lysosomal signaling that is indispensable for a properly functioning autophagic pathway.

### Loss of Gal-3 increases inflammasome activity and causes bacteria-induced inflammasome activation

**Figure 6:**
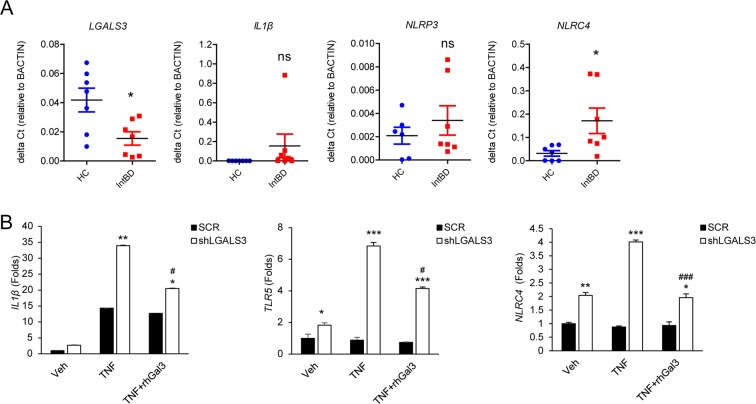
Galectin-3 affects inflammasomes in the colon tissues from patients with intestinal BD and intestinal epithelial cells. (**A**) mRNA levels in colon tissues. Transcript levels of *LGALS3, IL-1B, NLRP3*, and *NLRC4* from human colon tissues were quantified by real-time quantitative reverse transcription polymerase chain reaction and normalized to *β-actin*. **(B)** Gene expression related to inflammasomes (*IL-1B*, *NLRC4*, *TLR5***)** in cells treated with rhTNF-α or LPS. Data represent mean ± SD (n = 3). Cells were treated with TNF-α (40 ng/ml) or LPS (1 μg/ml) for 4 h in mRNA analysis and for 24 h in protein analysis. The transcript levels were quantified by real-time quantitative reverse transcription polymerase chain reaction and normalized to *β-actin*. ^*^p < 0.05 vs. HC or SCR, ^**^p < 0.01 vs. HC or SCR, ^***^p < 0.005 vs. HC or SCR, ^#^*p* < 0.05 vs. TNF-α, ^##^*p* < 0.01 vs. TNF-α, ^###^*p* < 0.005 vs. TNF-α. Veh, treated with phosphate-buffered saline; rhTNF-α, treated with recombinant human TNF-α; LPS, treated with lipopolysaccharide; rhGal3, treated with recombinant human galectin-3; SCR, scramble control HT-29 cells; shLGALS3, HT-29 cells expressing stably LGALS3 shRNA.

Autophagy negatively regulates inflammasome activity^32^ and accumulating evidence has shown that the inflammasome plays a pivotal role in host defense against intestinal microbes and intestinal inflammation^33^. Inflammasome activation results in the recruitment and activation of caspase-1, a key enzyme in the processing of pro-IL-1β into the mature IL-1β. This finding, together with our previous observations that autophagy is defective in cells lacking Gal-3, led us to determine whether *NLRP3, NLRC4*, and *IL-1B* transcript levels were altered in inflamed colon tissues of intestinal BD patients compared to non-affected control tissues. As shown in Fig. 6A, *NLRP3* mRNA levels were not significantly different between inflamed and control tissues, whereas expression of *IL-1B* (though statistically not significant) and *NLRC4* were higher in inflamed colon tissues compared to healthy controls. Consistently, *IL-1B*, *NLRC4*, and *TLR5* mRNA levels were significantly higher in shLGALS3 HT-29 cells compared to scramble shRNA-transfected cells (Fig. 6B).

**Figure 7:**
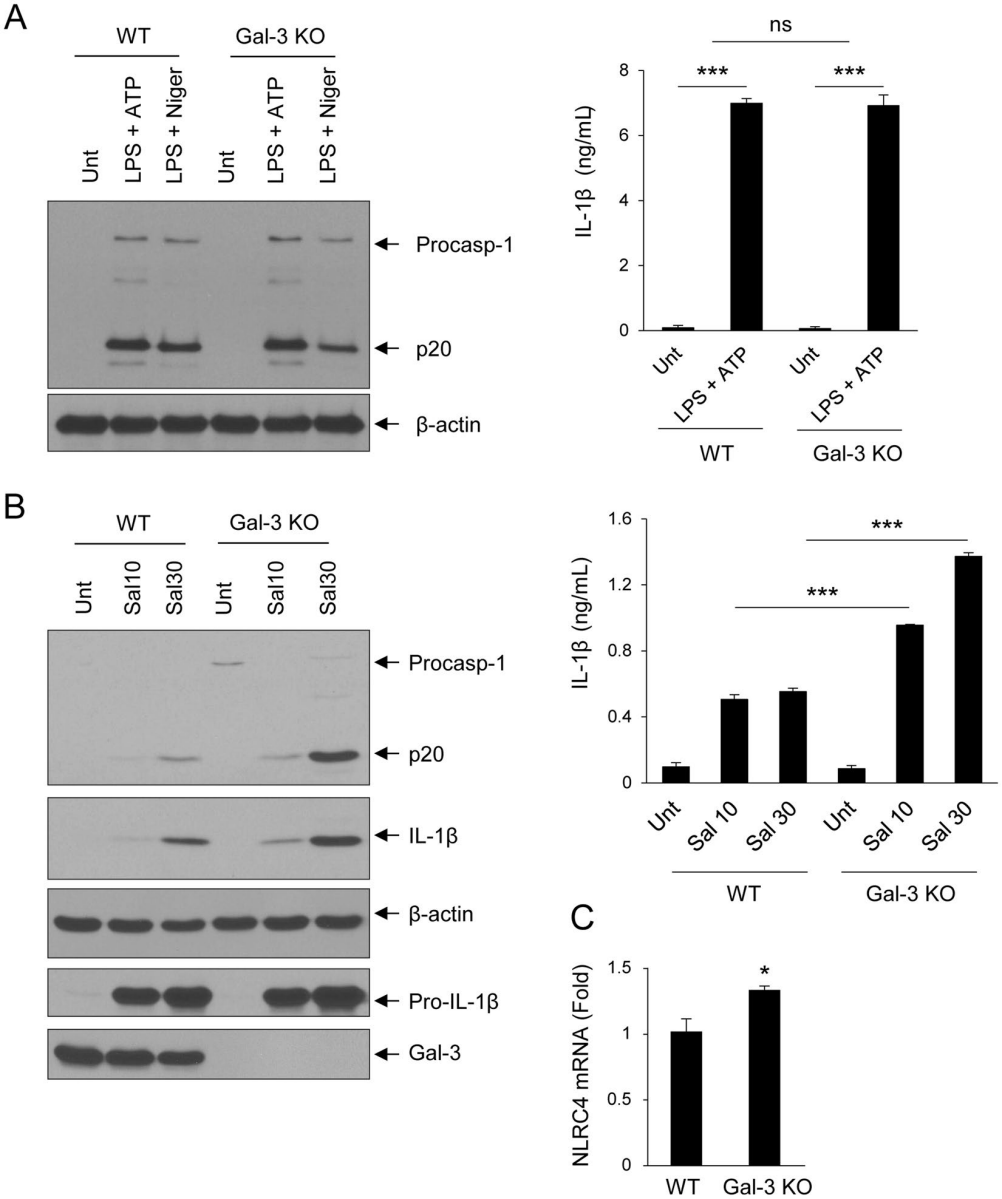
Loss of galectin-3 increases NLRC4 inflammasomes in macrophages. (**A)** Bone marrow-derived macrophages (BMDMs) were left untreated or treated with LPS, ATP, or nigericin. Cultural supernatants or soluble lysates were immunoblotted with anti-caspase 1. IL-1β levels in culture media were measured by enzyme-linked immunosorbent assay (ELISA). **(B)** BMDMs were infected with *S*. *typhimurium*. Supernatants or lysates were immunoblotted with the appropriate antibodies, as indicated. IL-1β levels in the culture media were measured by ELISA. **(C)** mRNA levels of *Nlrc4* in the colon tissues of wild-type (WT) and galectin-3 (Gal-3) knock-out (KO) mice were quantified by real-time quantitative reverse transcription polymerase chain reaction and normalized to *β-actin*. Unt, untreated; LPS treated with lipopolysaccharide; ATP, ATP-treated; Niger, nigericin-treated; Procasp-1, procaspase 1; p20, cleaved caspase 1; Pro-IL-1β, pro-interleukin 1 beta. *p < 0.05 vs. WT, ***p < 0.001 vs. untreated or WT.

**Figure 8:**
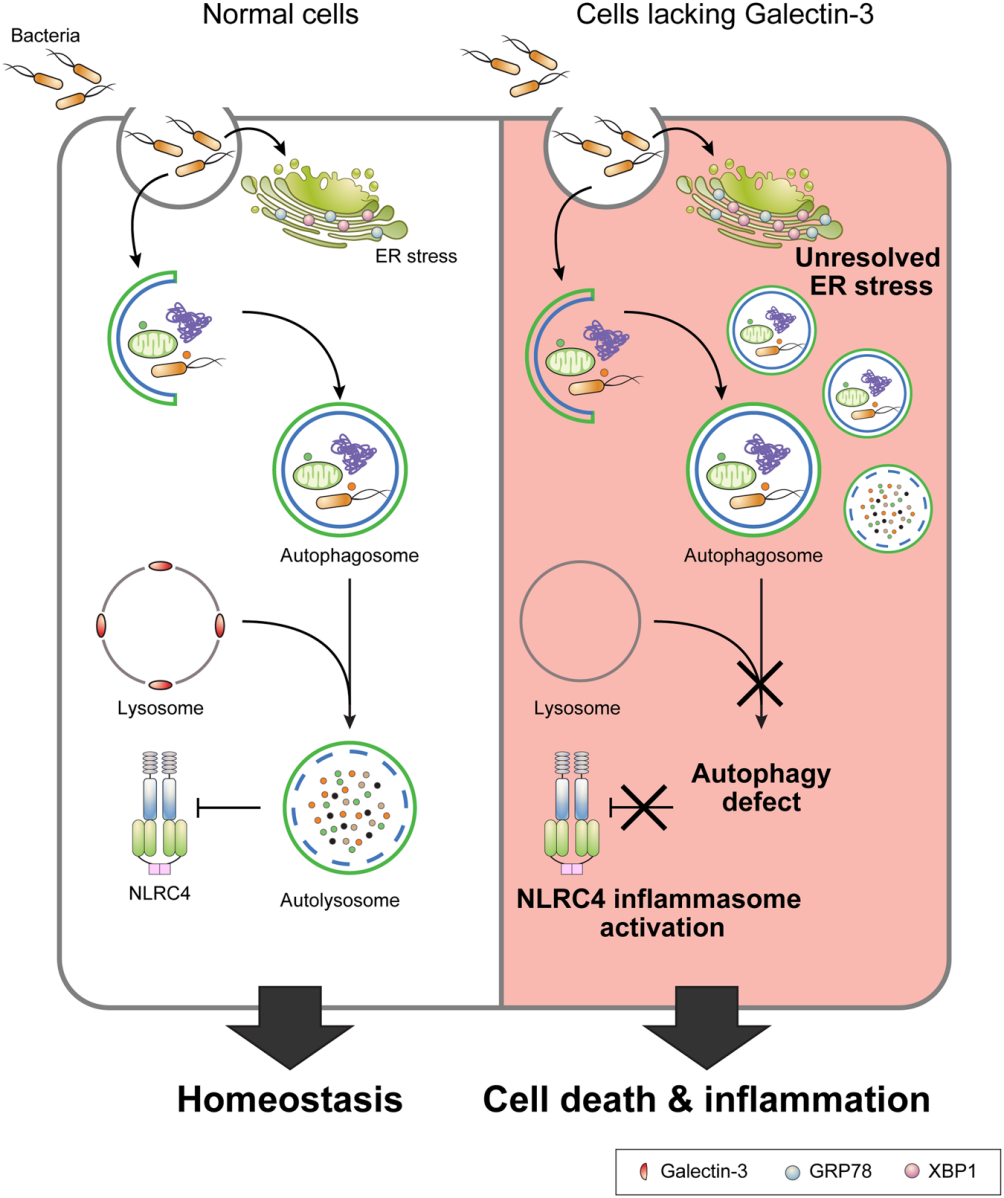
A model for galectin-3-mediated regulation of autophagy, endoplasmic reticulum (ER) stress, and inflammasomes in epithelial cells. Down-regulation of galectin-3 led to epithelial cell death due to unresolved ER stress and autophagy defects, resulting in activation of NLRC4 inflammasomes in response to autophagy defects.

Early control of host innate responses to bacterial infections is primarily dependent on macrophages, which function as the first line of host defense against a pathogen^34^. Therefore, we next sought to characterize the differential role of Gal-3 in NLRP3- or NLRC4-induced caspase-1 activation in macrophages using BMDMs from WT and *Lgal3*^−/−^ C57BL/6 mice. After BMDMs were treated with LPS plus adenosine triphosphate (ATP) or nigericin, a well described NLRP3 inflammasome stimulator, there was no significant difference in caspase-1 activation or IL-1β production (Fig. 7A and Supplemenatry Fig. 8A,B). However, infection with *Salmonella typhimurium*, activator of the NLRC4 inflammasome, led to caspase-1 activation and increased IL-1β production in BMDMs from *Lgal3*^−/−^ mice (Fig. 7B and Supplementary Fig. 8C–G). Importantly, *NLRC4* mRNA levels were higher in *Lgal3*^−/−^ BMDMs than in WT BMDMs (Fig. 7C). These results suggest that Gal-3 may act as a negative regulator of NLRC4, but not NLRP3.

## Discussion

In the present study, we utilized a proteomics approach to identify a distinct protein profile in intestinal tissues from intestinal BD patients. Among other differential protein levels, we observed significantly lower Gal-3 levels in intestinal BD colon tissues, an interesting observation given that Gal-3 has been reported as an immune response regulator in other chronic inflammatory disorders, including IBD. Using patient tissues, HT-29 cells, and murine BMDMs, we demonstrated that Gal-3 modulates ER stress, autophagy, and inflammasome activation, which suggests that the Gal-3 protein may play a protective role against intestinal BD. To the best of our knowledge, this is the first study to identify and characterize a biomarker of intestinal BD using a proteomics approach.

IBD is thought to be caused by an inappropriate and continuing inflammatory response to commensal microbes and epithelial barrier defects in a genetically susceptible host. To date, GWAS successfully identified genetic loci that contribute to IBD pathogenesis, including genes encoding barrier function proteins (GNA12 and HNF4A), innate and adaptive immune regulatory proteins (NOD2, CARD9, IL23R, and TNFSF15), autophagy proteins (ATG16L1, IRGM, and LRRK2), and ER stress proteins (XBP1)^10,11,21^. However, studies in the context of IBD have shown that genetic changes do not always translate directly into protein activity, and importantly, genomics does not consider posttranslational modifications. Therefore, in recent decades, proteomics approaches have proven a complementary method to genomics for biomarker discovery in IBD^14–16^. Some markers have been revealed that differentiate IBD subtypes (serum antibody anti-*Saccharomyces cerevisiae* antibody; ASCA, perinuclear anti-neutrophil cytoplasmic antibody; pANCA)^35,36^, while others predict relapse (fecal calprotectin)^23^. Similar to IBD, intestinal BD is known to have a wax-and-wane disease course and frequent relapse, even with surgery^5^; however, data regarding disease pathogenesis are extremely limited. Because we recently reported that genetic variants in *IL17A*, *IL23R*, *STAT4, NAALADL2*, and *YIPF7* are associated with intestinal BD pathogenesis^12,13^, in this study, we aimed to investigate differentially expressed proteins of intestinal BD using a proteomics approach.

We initially identified seven highly differential protein spots in colonic tissues from intestinal BD patients, including proteins identified as heat shock proteins, proteins involved in oxidative stress, and inflammatory proteins^23–25^. Among them, in particular, we observed significantly lower levels of Gal-3 in inflamed colonic tissues from intestinal BD patients, which is in line with prior studies reporting reduced levels of the Gal-3 protein in inflamed IBD tissues^20,37,38^. We therefore hypothesized that Gal-3 plays a protective role in intestinal BD and sought to further elucidate the underlying mechanisms of Gal-3 in intestinal immune responses in inflammatory bowel conditions.

Gal-3, a 31-kDa chimeric lectin, is a member of a large family of S-type lectins characterized by binding affinity for β-galactose-containing carbohydrates. Gal-3, which can be found both intra- and extra-cellularly, is expressed by various cell types, including epithelial cells in the gastrointestinal tract and activated immune cells^39–41^. By binding to its glycan ligand at the cell surface, Gal-3 regulates inflammatory responses by influencing cytokine secretion, cell adhesion and migration, and apoptosis, and additionally controls immune responses by regulating damage-associated molecular patterns and pathogen-associated molecular pattern pathways^39–41^. Although Gal-3 is thought to play a critical role in the modulation of chronic inflammatory disorders such as IBD and other autoimmune diseases^26–28,42^, findings have been contradictory. For example, several studies suggest that Gal-3 negatively regulates inflammatory responses that diminish interaction between Gal-3 and laminin, potentially causing increased intestinal permeability^38^ and Gal-3 downregulation may result in inappropriate T cell proliferation^37^. In contrast, other studies have reported a pro-inflammatory role of Gal-3 that may increase cell survival^43^ and promote activation of the NLRP3 inflammasome in macrophages^42^. Our current findings strongly support a protective role of Gal-3 against the development of intestinal inflammation. Further studies comparing the expression levels of Gal-3 between intestinal BD and IBD tissues could clarify the distinctive pathogenic role of Gal-3 in intestinal BD.

Barrier integrity is important to maintain immune tolerance towards intestinal microbiota and prevent chronic intestinal inflammation^7,9^. Upon ER stress either by genetic or environmental factors, GRP78 senses misfolded proteins in the stressed ER and activates three distinct unfolded protein response (UPR) signaling pathways to reduce ER stress and induce autophagy^44,45^. In addition, both UPR (*XBP1* and *AGR2*) and autophagy (*ATG16L1*, *IRGM*, and *LRRK2*)-related genes have been associated with IBD^10,11^ and several studies have shown evidence for increased ER stress in the small intestine of CD patients^46^. Similarly, we demonstrate here that reduced levels of the Gal-3 protein in HT-29 cells were associated not only with increased *GRP78* expression and *XBP1* splicing, but also with increased LPS or serum starvation-induced cell death. These findings suggest that unresolved ER stress in Gal-3 knockdown intestinal epithelial cells might lead to increased apoptosis which ultimately disrupts epithelial barrier integrity.

Autophagy, a mechanism for the resolution of ER stress, is activated in response to multiple stresses including hypoxia, infection, and nutritional starvation. Thus, UPR inevitably engages autophagy to compensate for ER stress in the intestinal epithelia^9,45^. Because it was reported that Gal-3 might be used as a marker of damaged endomembranes^31^ and Gal-3 on damaged lysosome can be targeted by autophagy^47,48^, we evaluated whether loss of Gal-3 affected autophagic clearance in an ER stress condition. We noted that Gal-3 knockdown epithelial cells had fewer intact lysosomes compared to control cells, showing more ruptured or aberrant lysosomes. Accompanying LC3B accumulation, in turn, might be caused by impaired degradation of autophagosomes due to an autophagy defect, as shown in a previous study^49^. In addition, Gal-3 was colocalized with lysosomes, and not autophagosomes. Upon binding to its ligand, non-lectin domains of Gal-3 interact with one another and lead to oligomerization in lattices, which may serve as a platform for assembly of other proteins and the occurrence of signaling^31^. The lysed membrane labeled by Gal-3 indeed recruits autophagy markers and is degraded by autophagic pathways^48^. All these data indicate that uncompensated ER stress and autophagy defects in intestinal epithelial cells lacking Gal-3 promote intestinal inflammation and lead to cell death in inflammatory conditions^44,45^, exacerbating colitis^29,30^. Nevertheless, further studies are needed to establish a direct link between Gal-3, ER stress, autophagy, and the development of intestinal BD.

Autophagy also negatively regulates inflammasome activity by degrading and removing assembled inflammasomes, by removing damaged mitochondria that would otherwise promote inflammasome activation, and by downregulating pro-IL-1β^32^. We observed increased levels of caspase-1 activation, IL-1β production, and NLRC4 inflammasome activation in *Lgals3*^‒/‒^ BMDMs, consistent with a previous report that *ATG16L1*-deficient cells have enhanced inflammasome activation in response to TLR stimulation^50^. Our results suggest that autophagy defects in response to bacterial triggers in turn leave inflammasome activation unchecked and hyperactive.

It is intriguing that we observed a distinct effect of Gal-3 on the regulation of inflammasomes, in that Gal-3 appears to regulate NLRC4, but not NLRP3 inflammasomes. Previous studies have demonstrated that intestinal phagocytic cells can detect invading pathogenic bacteria by the NLRC4 inflammasome and trigger inflammatory responses^33,51^. Because the role of inflammasomes in colitis is not yet fully understood, further studies are needed to clarify the role of Gal-3-regulated inflammasomes in intestinal BD pathogenesis.

We additionally observed decreased *IL-10* levels in HT-29 cells transfected with shLGALS3. Consistent with this observation, reduced IL-10 expression in response to Gal-3 downregulation has been observed in dendritic cells^52^, further supporting a role for Gal-3 in downregulating immune responses that may incite intestinal inflammation. Because it has been also demonstrated that the disruption of IL-10 causes a loss of suppression of the mucosal immune response and even excessive apoptosis and necrosis^53^, loss of IL-10 in cells with reduced Gal-3 expression may aggravate intestinal inflammation and cell death. However, our results were not reproduced with rhGal-3 administration. Several studies have suggested a different role for Gal-3 depending on various factors such as target cells and specific inflammatory conditions^39^ implicating the need for further investigation into the regulation of T-cell function by Gal-3.

Here we describe for the first time a novel pathway by which Gal-3 down-regulation may play a protective role in the development of intestinal BD via ER stress, autophagy defects, and NLRC4 inflammasome activation (Fig. 8), suggesting new insights of Gal-3 in the intestinal BD disease pathogenesis. Further large-scale studies are warranted to confirm our findings. In addition, our data need to be replicated in a colitis model specific for intestinal BD. BD-like mouse model produced by herpes simplex virus inoculation showed BD-like symptoms including skin manifestations, eye symptoms, orogenital ulcers, and gastrointestinal ulcer (5.2%)^54^. Unfortunately, however, there are currently no specific animal models for intestinal BD. By unravelling the pathogenic mechanisms of intestinal BD, the diagnosis of intestinal BD is likely to be improved, leading to faster and more appropriate treatments for patients, ultimately increasing their quality of life.

## Supplementary information

Supplementary Information
