# Supplementary material for: Proteomics-based functional studies reveal that galectin-3 plays a protective role in the pathogenesis of intestinal Behçet’s disease
Source: Sci Rep. 2019 Aug 12;9:11716. doi: 10.1038/s41598-019-48291-1 (PMC6691011; doi:10.1038/s41598-019-48291-1)
Supplement: Supplementary file 1 — Supplementary Information [file 41598_2019_48291_MOESM1_ESM.pdf]

Proteomics-based functional studies reveal that galectin-3 plays a protective role in the pathogenesis of intestinal Behçet's disease

Hyun Jung Lee, M.D., Ph.D.[1,2*], Jae Hyeon Kim[1,3*], Sujeong Hong[4], Inhwa Hwang[4], Soo Jung Park, M.D., Ph.D.[1], Tae Il Kim, M.D., Ph.D.[1], Won Ho Kim, M.D, Ph.D.[1], Je-Wook Yu, Ph.D.[4], Seung Won Kim, Ph.D.[1,3,5†], Jae Hee Cheon, M.D, Ph.D.[1,3,5†]

[1]*Department of Internal Medicine and Institute of Gastroenterology, Yonsei University College of Medicine, Seoul, Korea*

[2]*Department of Internal Medicine and Liver Research Institute, Seoul National University College of Medicine, Seoul, Korea*

[3]*Brain Korea 21 PLUS Project for Medical Science, Yonsei University College of Medicine, Seoul, Korea*

[4]*Department of Microbiology, Institute for Immunology and Immunological Diseases, Brain Korea 21 PLUS Project for Medical Science, Yonsei University College of Medicine, Seoul, Korea*

[5]*Severance Biomedical Science Institute, Yonsei University College of Medicine, Seoul, Korea*

*,†These authors contributed equally to this work

Corresponding authors:

Jae Hee Cheon, MD, PhD, Department of Internal Medicine, Yonsei University College of Medicine, 50-1 Yonsei-ro, Seodaemun-gu, Seoul 03722, Korea. GENIUSHEE@yuhs.ac;

Seung Won Kim, PhD, Department of Severance Biomedical Science Institute, Yonsei University College of Medicine, 50-1 Yonsei-ro, Seodaemun-gu, Seoul 03722, Korea.

swk21c@hanmail.net

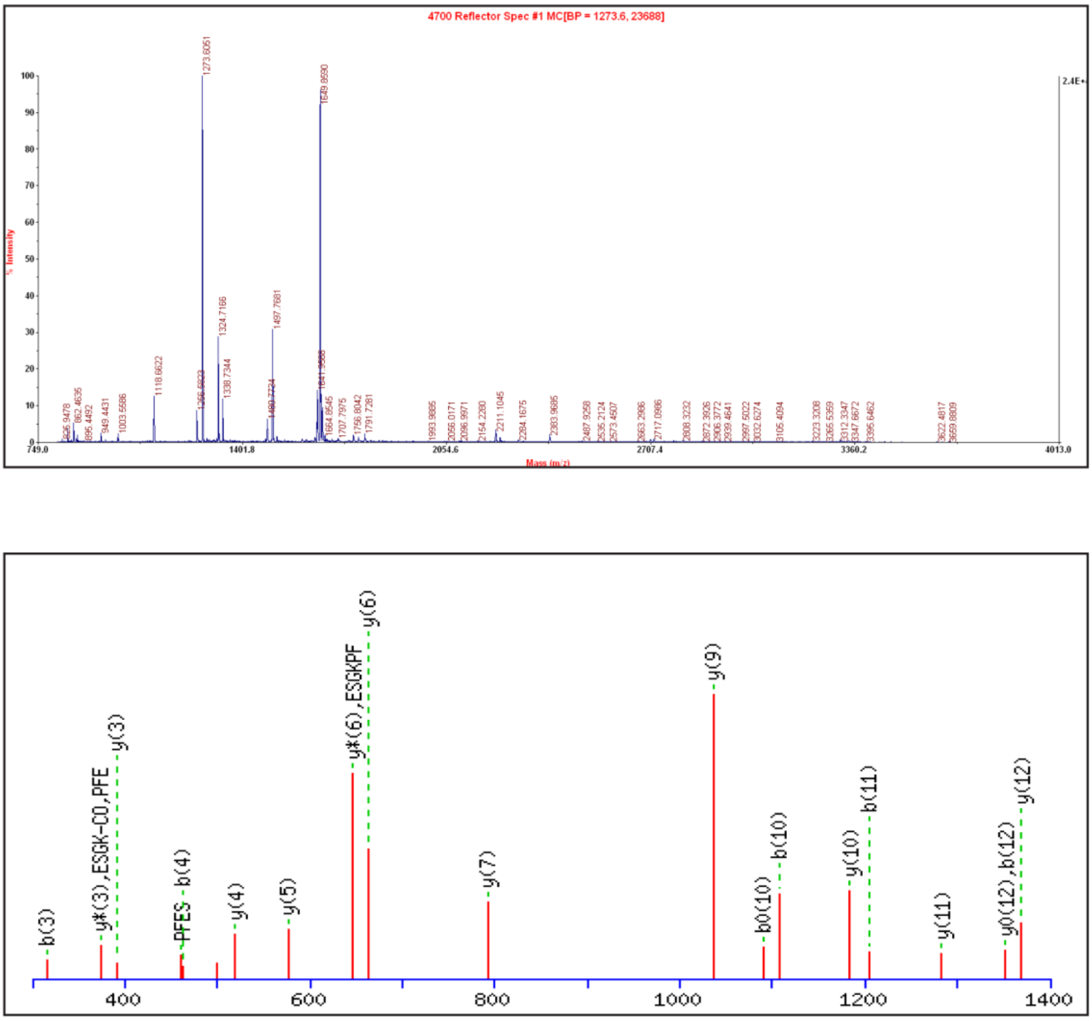

Supplementary Figure 1. Identification of galectin-3 by peptide mass fingerprinting (PMF) and matrix-assisted laser desorption/ionization tandem time-of-flight mass spectrometry (MS/MS). Typical PMF and MS/MS spectrum for the galectin-3 (Gal-3) protein.

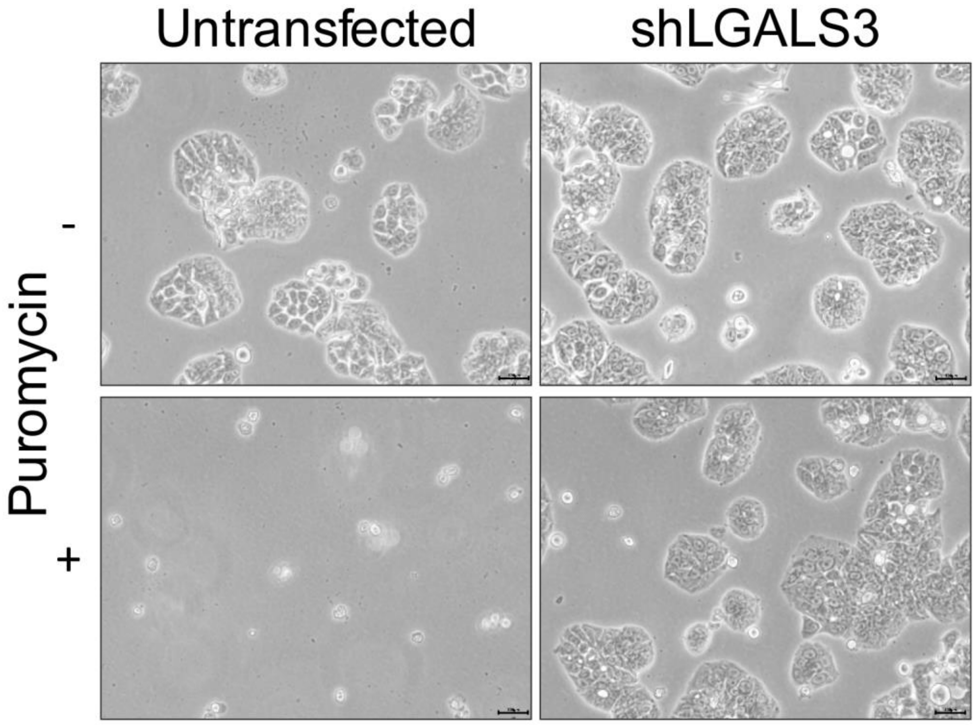

Supplementary Figure 2. Establishment of a stable cell line. We transfected HT-29 cells with human Gal-3 shRNA plasmids (shLGALS3) to create Gal-3 knock-down cell lines. Successfully transfected cells were isolated by selection with 20 μg/ml puromycin for 4 weeks. Images were taken with a light microscope at 400× magnification.

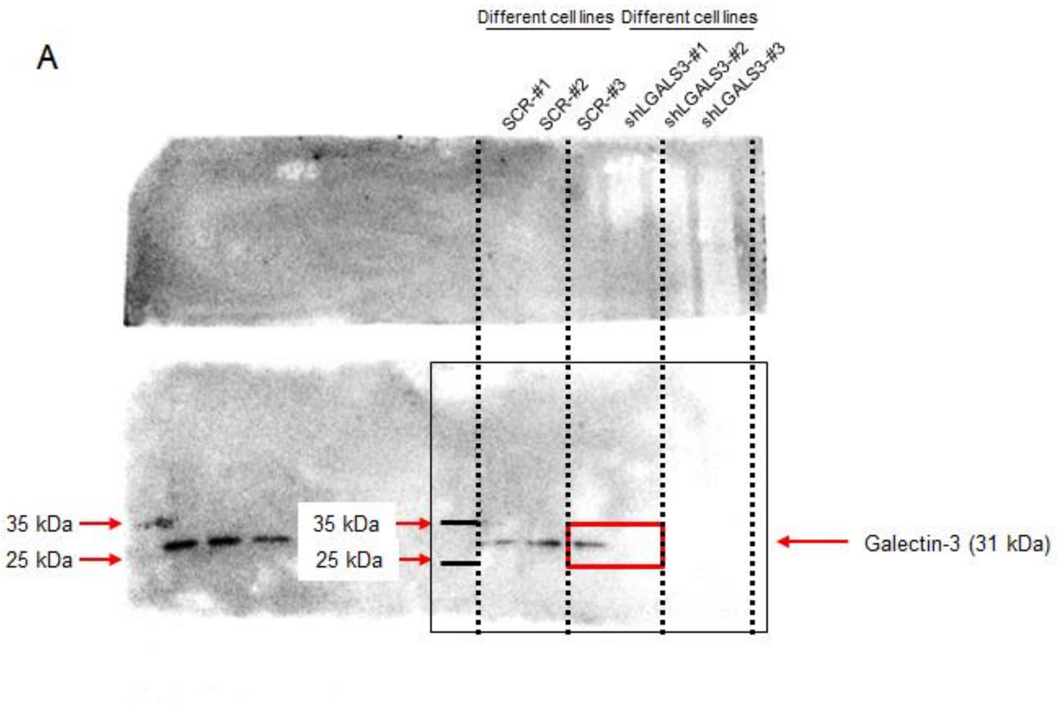

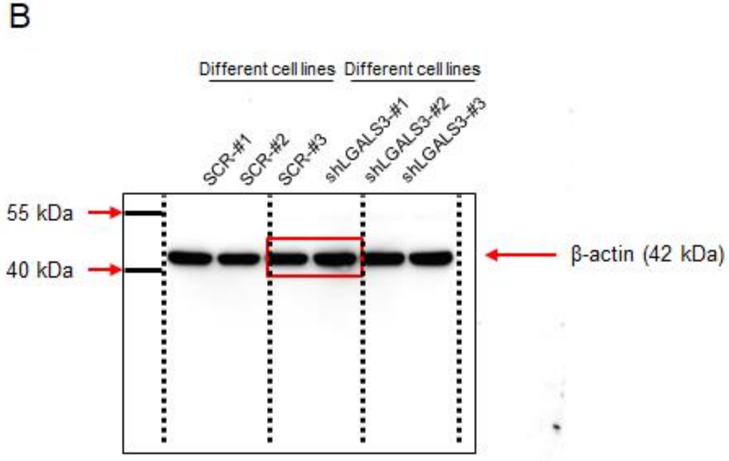

Supplementary Figure 3. Confirmation of a stable cell line. Protein levels of Galectin-3 **(A)** and *β-actin* **(B)**. SCR, scramble control HT-29 cell; shLGALS3, HT-29 cell stably expressing LGALS3 shRNA.

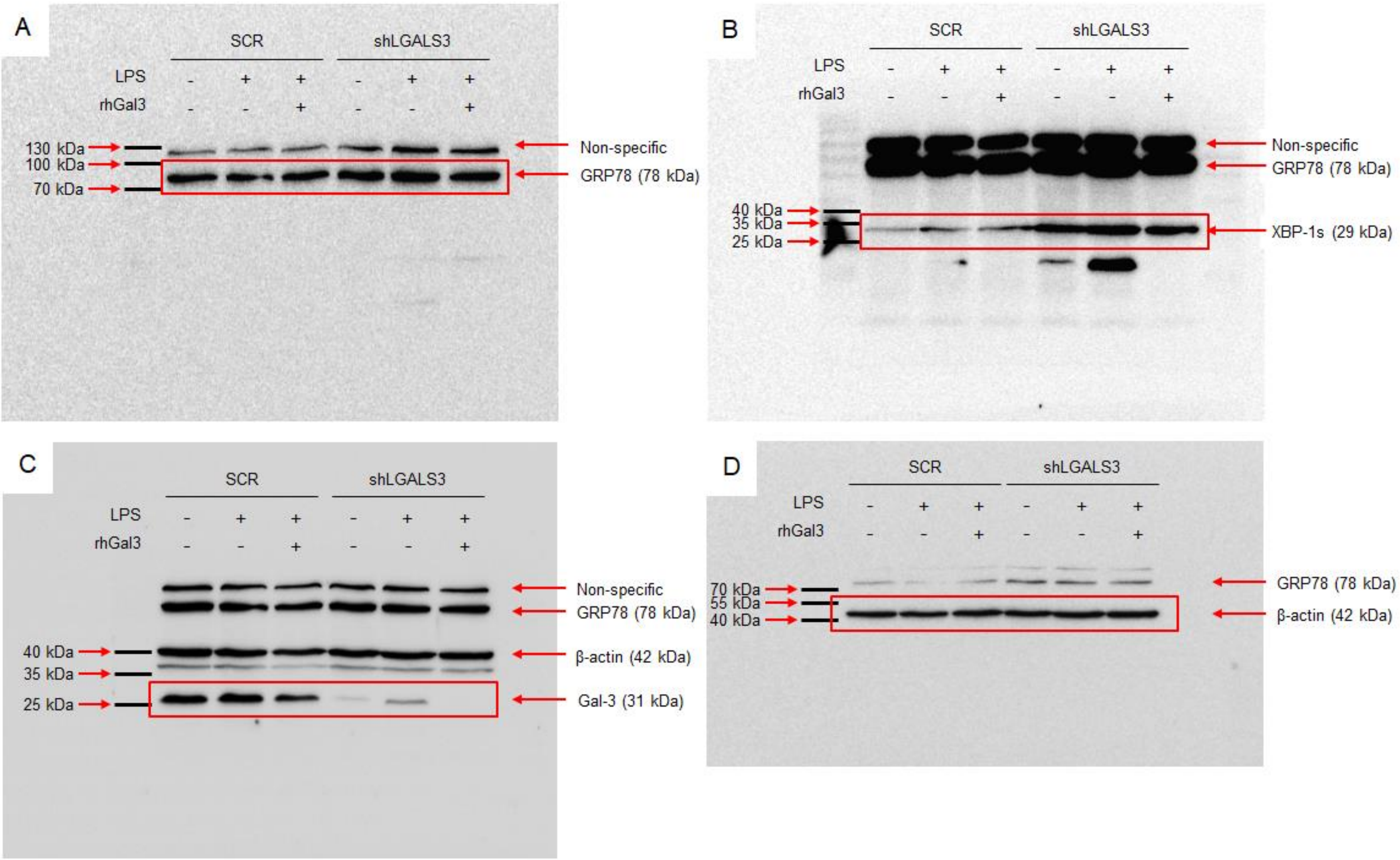

Supplementary Figure 4. Loss of galectin-3 activates endoplasmic reticulum (ER) stress. Protein levels of GRP78 **(A)**, XBP-1s **(B)**, Galectin-3 **(C)**, and *β-actin* **(D)**.

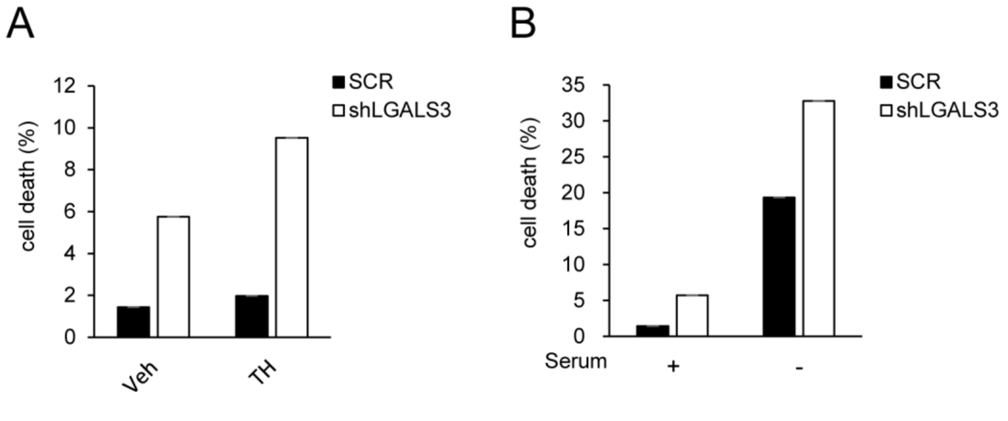

Supplementary Figure 5. Loss of galectin-3 leads to endoplasmic reticulum (ER) stress-induced cell death. Effects of galectin-3 on cell death by an ER stress inducer **(A)** and serum starvation (**B**). Representative data of Annexin V/PI staining. Cells were treated with thapsigargin (an ER-stress inducer) or in serum-free conditions for 24 h.

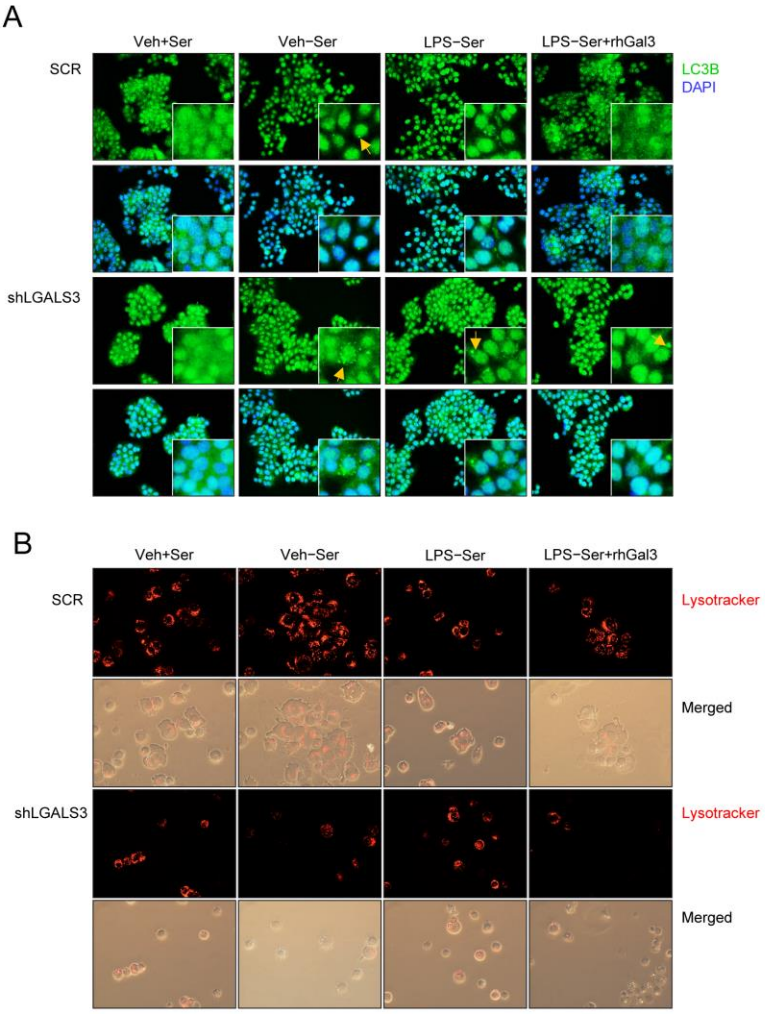

Supplementary Figure 6. Galectin-3 knockdown induces abnormal autophagosome and lysosome function. (A) Representative images of LC3B and DAPI stained cells (green and blue, respectively). Yellow arrows indicate the punctate pattern of LC3B in autophagic cells. **(B)** Representative images of lysosomes. Red color indicates LysoTracker. Images were taken with a fluorescence microscope at 400× magnification. Experiments were performed in triplicate. Veh, treated with phosphate-buffered saline; +Ser, 10% serum supplemented; -Ser, serum free; LPS, treated with lipopolysaccharide; rhGal3, treated with recombinant human galectin-3 (10 μM); SCR, scramble control HT-29 cells; shLGALS3, HT-29 cells stably expressing LGALS3 shRNA.

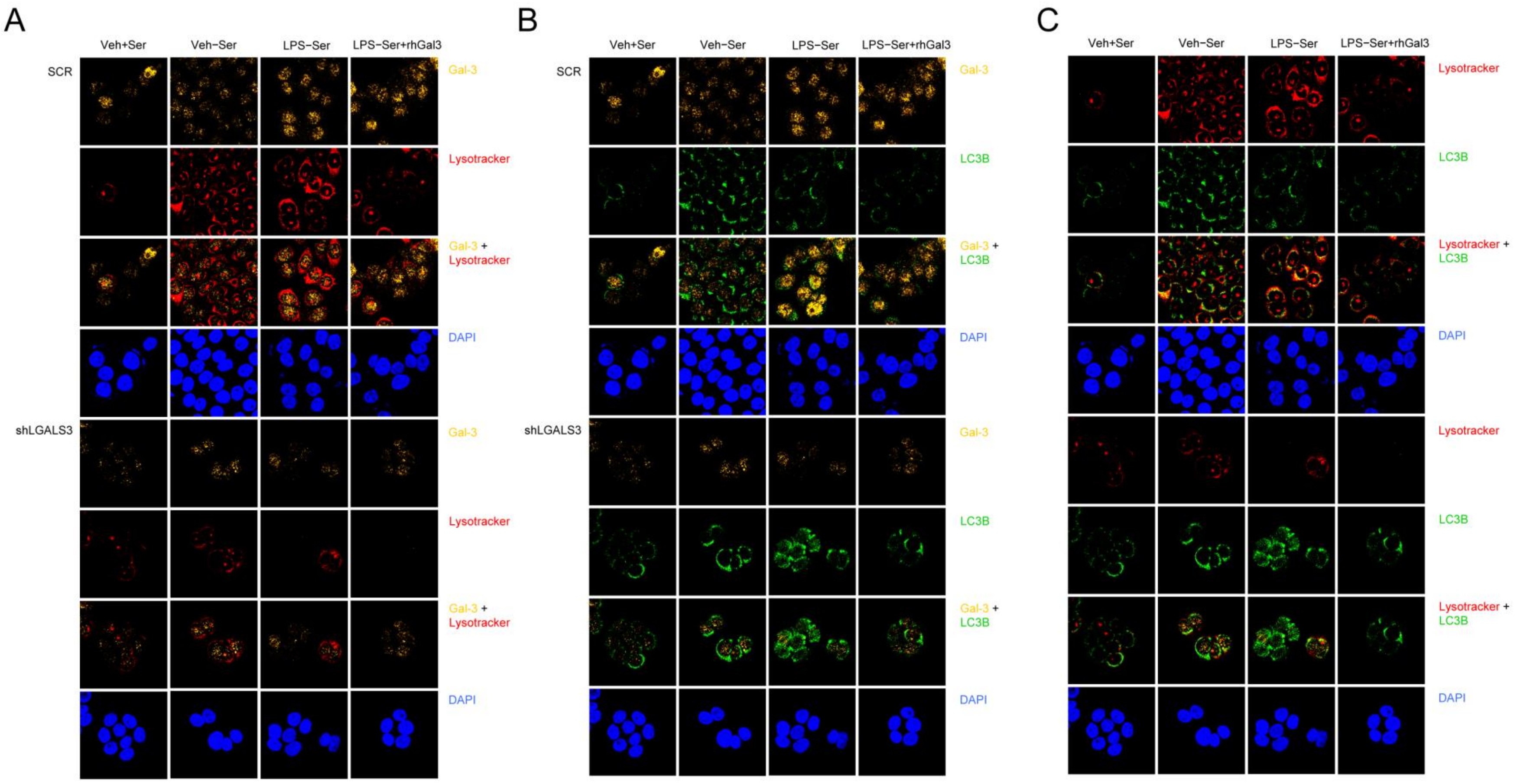
A
Veh+Ser
Veh−Ser
LPS−Ser
LPS−Ser+rhGal3
SCR
Gal-3
Lysotracker
Gal-3 + Lysotracker
DAPI
shLGALS3
Gal-3
Lysotracker
Gal-3 + Lysotracker
DAPI
B
Veh+Ser
Veh−Ser
LPS−Ser
LPS−Ser+rhGal3
SCR
Gal-3
LC3B
Gal-3 + LC3B
DAPI
shLGALS3
Gal-3
LC3B
Gal-3 + LC3B
DAPI
C
Veh+Ser
Veh−Ser
LPS−Ser
LPS−Ser+rhGal3
Lysotracker
LC3B
Lysotracker + LC3B
DAPI
Lysotracker
LC3B
Lysotracker + LC3B
DAPI

Supplementary Figure 7. Galectin-3 knockdown induces autophagy defects. (A) Representative confocal images of galectin-3 (Gal-3) and LyzoTracker localization. **(B)** Representative confocal images of Gal-3 and LC3B localization. **(C)** Representative confocal images of LC3B and LyzoTracker localization. All images were obtained with a confocal microscope at 800× magnification. Veh, treated with phosphate-buffered saline; +Ser, 10% serum supplemented; -Ser, serum-free; LPS, treated with lipopolysaccharide; rhGal, treated with recombinant human galectin-3 (10 μM); SCR, scramble control HT-29 cells; shLGALS3, HT-29 cells expressing stably LGALS3 shRNA.

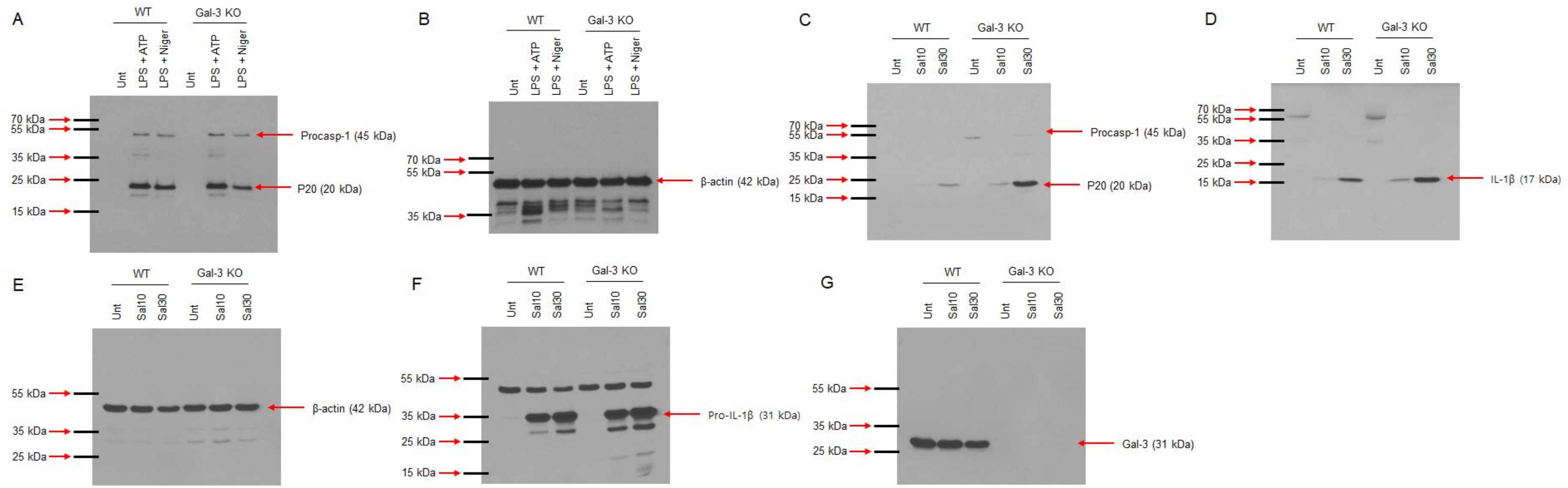

Figure 8. Loss of galectin-3 increases NLRC4 inflammasomes in macrophages. (A, B) Protein levels of caspase-1 **(A)** and *β-actin* **(B)**. Bone marrow-derived macrophages (BMDMs) were untreated or treated with LPS, ATP, or nigericin. **(C–G)** Protein levels of caspase-1 **(C)**, IL-1β **(D)**, *β-actin* **(E)**, pro-IL-1β **(F)**, and Galectin-3 **(G)**. BMDMs were infected with *S. typhimurium*.

Supplementary Table 1. Nucleotide sequences of primers used in the study

	Forward	Reverse
Human primers		
LGALS3	CAATACAAAGCTGGATAATAACTGG	GATTGTACTGCAACAAGTGAG
IL1B	AGCTACGAATCTCCGACCAC	CGTTATCCCATGTGTCGAAGAA
IL10	TTACCTGGAGGAGGTGATGC	GGCCTTGCTCTTGTTTTCAC
TGFB	AAGGACCTCGGCTGGAAGTG	CCGGGTTATGCTGGTTGTA
NLRP3	CGGGGCCTCTTTTCAGTTCT	CCCCAACCACAATCTCCGAA
NLRC4	TCAGAAGGAGACTTGGACGAT	GGAGGCCATTCAGGGTCAG
GRP78	AGTTCTTGCCGTTCAAGGTG	AGACCGGAACAGATCCATGT
TLR5	TTCAACTTCCCAAATGAAGGA	TTGCATCCAGATGCTTTTCA
XBP1s	TCTGCTGAGTCCGCAGCAG	GAAAAGGGAGGCTGGTAAGGAAC
β-ACTIN	CTCTTCCAGCCTTCCTTCCTG	CAGCACTGTGTTGGCGTACAG
Mouse primers		
Nlrc4	GAAACACTGTACGATCAGCTCC	CATGTTCTTGAAGCGATGGTTTT
β-ACTIN	AGTGTGACGTTGACATCCGT	TGCTAGGAGCCAGAGCAGTA

Supplementary Table 2. Characteristics of controls and patients with intestinal Behçet's disease

	Control	Intestinal BD
No. of patients	17	17
Male/female	6/11	8/9
Age (yrs, mean ± SD)	46.01 ± 10.24	53.23 ± 9.02
Disease duration (months)	NA	115.52 ± 52.79
Indication of surgery (%)		
Intestinal perforation	NA	5 (29.4)
Intractability to medical treatments	NA	12 (70.6)
Colorectal cancer	15 (88.2)	NA
Malignant bowel obstruction due to stomach cancer	1 (5.9)	NA
Others	1 (5.9)[a]	NA
Surgery type (%)		
Ileocecectomy	NA	13 (76.5)
Segmental resection of small bowel	NA	1 (5.9)
Right hemicolectomy	16 (94.1%)	2 (11.8)
Others	1 (5.9)[b]	1 (5.9)[c]

Int BD, intestinal Behçet's disease; NA, not applicable
[a]Appendiceal mucocele; [b]Anterior resection; [c]Total colectomy
